# Trends in indirect liver function marker testing in Wales from 2000 to 2017 and their association with age and sex: an observational study

**DOI:** 10.1136/bmjgast-2022-000885

**Published:** 2022-04-29

**Authors:** Trevor Alexander Hill, Colin John Crooks, Joe West, Joanne R Morling

**Affiliations:** 1 Translational Medical Sciences, NIHR Nottingham Biomedical Research Centre (BRC), School of Medicine, University of Nottingham, Nottingham, UK; 2 Population and Lifespan Sciences, NIHR Nottingham Biomedical Research Centre (BRC), School of Medicine, University of Nottingham, Nottingham, UK

**Keywords:** screening, liver cirrhosis, epidemiology, liver function test

## Abstract

**Objective:**

If non-invasive markers of liver fibrosis were recorded frequently enough in clinical practice, it might be feasible to use them for opportunistic community screening for liver disease. We aimed to determine their current pattern of usage in the national primary care population in Wales.

**Design:**

Using the Secure Anonymised Information Linkage (SAIL) Databank at Swansea University (2000–2017), we quantified the frequency of common liver blood tests (aspartate aminotransferase (AST), alanine aminotransferase (ALT), platelet count and albumin) used in fibrosis marker algorithms. We examined measurement variation by age and sex.

**Results:**

During the 18-year study period, there were 2 145 178 adult patients with at least one blood test available for analysis. Over the study period, the percentage of SAIL patients receiving an ALT test in each year increased from 2% to 33%, with platelet count and albumin measurement increasing by a similar factor. AST testing, although initially rising, had decreased to 1% by the end of the study. AST and ALT values varied by age and sex, particularly in males with the upper normal range of ALT values decreasing rapidly from 90 U/L at age 30 to 45 U/L by age 80.

**Conclusion:**

The reduction in AST testing to only 1% of the adult population limits the use of many non-invasive liver marker algorithms. To enable widespread screening, alternative algorithms for liver fibrosis that do not depend on AST should be developed. Liver fibrosis markers should be modified to include age-specific and sex-specific normal ranges.

**Key messages**

**What is already known on this topic**

- Liver disease is increasing worldwide.
- To identify individuals at risk of progressing to advanced and serious liver disease requires non-invasive liver fibrosis markers to screen the population that are cheaper and safer than other methods such as biopsy.

**What this study adds**

In the Welsh population:

- Alanine aminotransferase (ALT) and full blood count tests are being used more frequently.
- Aspartate aminotransferase (AST) testing has reduced.
- ALT and AST levels used in non-invasive fibrosis markers are dependent on age and sex.

**How this study might affect research, practice and/or policy**

- Due to the decrease in AST testing, widespread screening using existing fibrosis markers may not be possible. Alternative markers that do not depend on the AST should therefore be developed. Existing markers should also be modified to account for the age and sex of the subject.

## Introduction

Liver disease is on the increase, both worldwide and in the European Union.^1–3^ A recent review found that cirrhosis and liver cancer are, respectively, the 11th and 16th leading causes of death globally, and worldwide they account for 3.5% of all deaths.^1^ There is growing consideration of the use of non-invasive liver fibrosis markers in population screening as an approach to this problem.^4 5^

An earlier diagnosis has the potential to save lives and money, as disease progression can be halted and fibrosis can be reversed.^6^ However, invasive techniques such as biopsy require a hospital visit, can be painful and in a few cases can be fatal.^6 7^ Conventional imaging techniques such as ultrasound and MRI can be affected by factors such as whether the patient is obese and are prone to interrater inconsistency. Although newer methods such as elastography and the use of contrast agents give the potential for improved diagnostic performance,^8 9^ they still require a hospital visit and their cost would make application to screening impractical.

There is therefore a need for a non-invasive, cheap alternative for the identification of advanced liver disease, which can be carried out repeatedly in a short time without complications.^10^ One option is to use blood serum tests of indirect liver markers that are frequently measured in primary care.^11^ To improve the diagnostic accuracy of these indirect markers, such as alanine aminotransferase (ALT) and aspartate aminotransferase (AST), various composite markers combine these with other factors, for example, the AST to platelet ratio index,^12^ AST/ALT ratio,^13^ fibrosis-4 index^14^ and non-alcoholic fatty liver disease (NAFLD) fibrosis score.^15^ Each of these was originally developed in a very specific population, but their uses have increased in recent years. To be useful on a population level for opportunistic screening for liver disease, they need to be recorded frequently enough as part of routine clinical care and their normal ranges must be relevant to the population being tested.

The aim of this study was to investigate and explore the current pattern of liver blood testing in the national primary care population in Wales, to assess the feasibility of routine screening for liver disease using the opportunistic recording of one or more of these non-invasive composite markers.

## Methods

### Study design

This retrospective, observational cohort study uses routinely collected blood test data from Welsh patient visits to their general practitioner (GP). The analysis is descriptive, and apart from presenting numbers aims to describe rates of testing over time.

### Population

The study cohort was identified from data stored on the Secure Anonymised Information Linkage (SAIL) Databank at Swansea University.^16 17^ We used the Welsh Longitudinal General Practice dataset for the period January 2000 to December 2017, which includes 77% of Welsh general practices accounting for 79% of Welsh patients and has been shown to be representative of the Welsh population in terms of sex, age and deprivation.^18^ All adults (≥18 years) were included for analysis. Patients with inconsistencies in their data, such as changes in sex or date of birth across practices were identified by linking the GP event and practice files using the anonymised linking field provided by SAIL,^19^ and were excluded.

### Liver blood tests

We identified all patients with one or more Read codes (online supplemental table S1) for liver-related blood serum tests for platelet count, serum albumin, ALT and/or AST in the analysis. Where an individual had multiple blood tests on the same day the mean value was used. Test data were excluded if there were multiple tests on the same day and the difference between them was outside the IQR for all the data, or the result contained missing, non-integer or extreme, implausible values (online supplemental figure S1).

10.1136/bmjgast-2022-000885.supp1 Supplementary data

**Figure 1:**
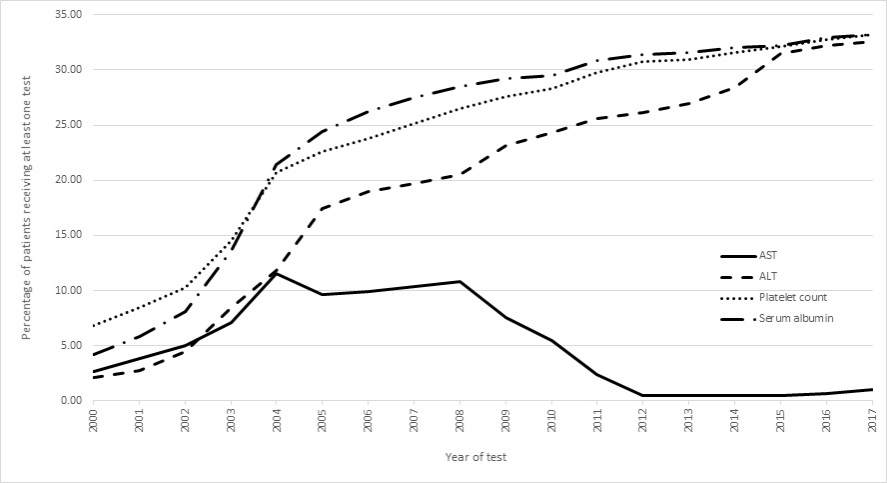
Percentage* of SAIL patients receiving each test for each year of the study. ALT, alanine aminotransferase; AST, aspartate aminotransferase; SAIL, Secure Anonymised Information Linkage. * The percentages for each year are generated as follows: The denominator is the total number of SAIL patients in that year for whom GP data was available. The numerator is the total number of study participants in that year who had that particular test. The figure is interpreted as follows: in 2011, 2.4% of SAIL patients received an AST test, whereas 26% of SAIL patients in the same year received the ALT test.

### SAIL denominator data

Total Welsh population estimates were determined for all adults aged at least 18 years for whom SAIL data was available during the study period, including estimates by calendar year, age band and sex.

### Analysis

For each liver blood test type, the number and prevalence of patients receiving one or more blood tests in each year, was calculated. The trends over time were examined, stratified by sex and age bands (18–39, 40–59, 60–79 and 80+ years). We also examined the age and sex dependency of ALT and AST by plotting the trend in median and upper 95th percentiles by sex, from ages 18 to 100 years.

## Results

### Patient numbers and characteristics

During the 18-year study period, there were 2 145 178 adult patients (representing 66.6% of the SAIL population) with at least one liver blood test available for analysis, associated with 40 077 685 blood test records. The most commonly available test result was platelet count (2 077 341 patients, 64.5% of all SAIL patients), followed by serum albumin (1 965 392, 61.0%), ALT (1 779 291, 55.2%) and AST (654 775, 20.3%).

The total monthly number of test results ranged from 20 594 in January 2000 to 204 066 in December 2017 (see online supplemental table S2 and figure S2 for monthly frequencies and percentages for all years combined). The total number of patients tested ranged from 121 728 in 2000 to 793 608 in 2017 (online supplemental table S3 and figure S3). Online supplemental figure S4 shows the final distributions for each test type.

**Figure 2:**
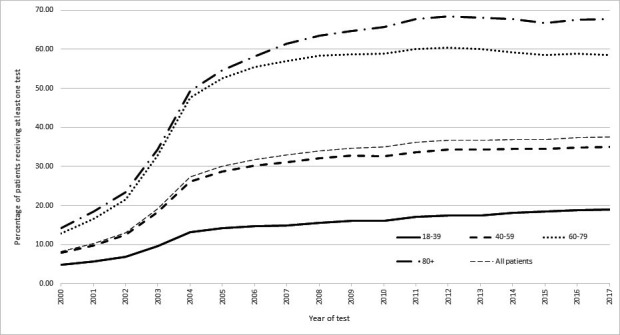
Percentage* of SAIL patients in each age band when tested, for each year of the study. SAIL, Secure Anonymised Information Linkage. * The percentages for each year are generated as follows: The denominator is the total number of SAIL patients in that year and age band for whom GP data was available. The numerator is the total number of study participants in each age band, who received any LFT test. The figure is interpreted as follows: in 2004, 13% of SAIL patients aged 18-39 were tested, 26% of those aged 40-59 were tested, 48% of those aged 60-79 were tested and 49% of those aged 80+ were tested.

**Figure 3:**
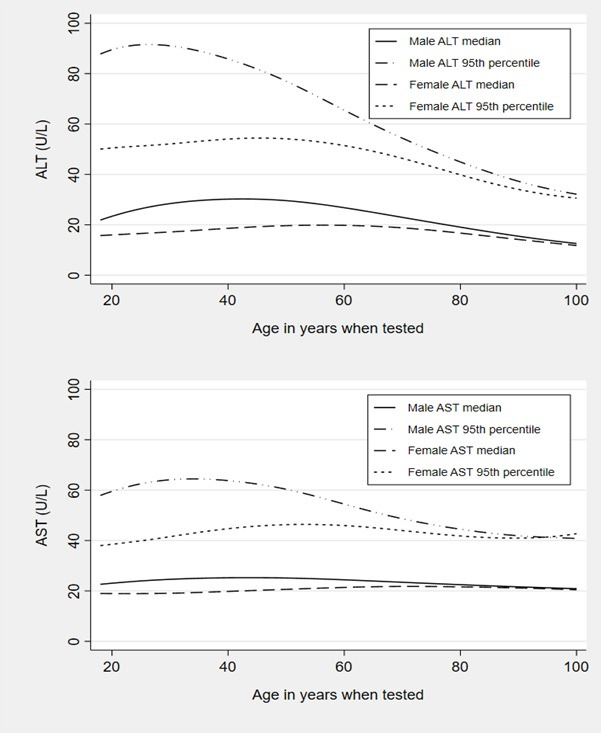
Median and 95^th^ percentile ALT and AST test scores by sex, ages 18–100. ALT, alanine aminotransferase; AST, aspartate aminotransferase.

There were 951 926 men (60.0% of male SAIL patients) and 1 193 252 women (72.9% of female SAIL patients) with at least one blood test. The age when tested ranged from 18 to >100 with a median (IQR) of 60 (45–72) years. Table 1 summarises the patient characteristics overall and by sex and age band.

**Table 1:** Patient characteristics

	All patients	Males	Females
No patients receiving each blood serum test*			
FBC (Platelet count)	2 077 341 (64.48%)	909 865 (57.43%)	1 167 476 (71.29%)
LFT (SA/ALT/AST)	1 971 370 (61.19%)	893 528 (56.40%)	1 077 842 (65.82%)
SA	1 965 392 (61.00%)	890 400 (56.20%)	1 074 992 (65.64%)
ALT	1 779 291 (55.23%)	804 222 (50.76%)	975 069 (59.54%)
AST	654 775 (20.32%)	293 036 (18.50%)	361 739 (22.09%)
No in analysis (any test)	2 145 178 (66.58%)	951 926 (60.01%)	1 193 252 (72.86%)
Total SAIL patients	3 221 889 (100.00%)	1 584 215 (100.00%)	1 637 674 (100.00%)
No blood serum tests†			
Platelet count	13 638 831 (34.03%)	5 339 278 (31.14%)	8 299 553 (36.19%)
Serum albumin	13 271 457 (33.11%)	5 899 390 (34.41%)	7 372 067 (32.14%)
ALT	10 656 162 (26.59%)	4 775 449 (27.86%)	5 880 713 (25.64%)
AST	2 511 235 (6.27%)	1 129 442 (6.59%)	1 381 793 (6.03%)
Total	40 077 685 (100.00%)	17 143 559 (100.00%)	22 934 126 (100.00%)
Median (IQR) test value‡			
Platelet count (×10^9^ /L)	259 (216–309)	238 (199–284)	272 (229–322)
SA (g/L)	42 (39–45)	43 (40–45)	42 (39–44)
ALT (U/L)	21 (15–31)	25 (18–36)	19 (14–26)
AST (U/L)	22 (19–28)	24 (20–30)	21 (18–26)
Age when tested§			
18–39	782 777 (43.83%)	299 318 (33.58%)	483 459 (54.06%)
40–59	901 262 (67.59%)	422 631 (61.91%)	478 631 (73.55%)
60–79	803 963 (80.69%)	388 530 (79.63%)	415 433 (81.70%)
80+	313 012 (80.81%)	123 163 (80.52%)	189 849 (81.00%)
Median (IQR) test age	60 (45–72)	62 (49–72)	59 (42–72)
Time spent in study¶			
Median (IQR) years	6 (1–12)	6 (0–11)	7 (1–12)

*Treating platelet count tests as separate venous samples, but ALT, AST and serum albumin tests on the same day as coming from the same venous sample. Percentages are with respect to the total number of SAIL patients.

†The number of blood test records of each type remaining after data cleaning.

‡Includes all blood test data, so some patients are included more than once.

§Patients are counted once within each age band they are tested in, so the sum is greater than the total number of patients in the study. Note that the median test age is derived slightly differently by including one patient per year when tested.

¶Years between first and last blood test from 2000 to 2017. A zero means the patient enters and leaves the study in the same year.

ALT, alanine aminotransferase; AST, aspartate aminotransferase; FBC, full blood count; LFT, liver function test; SA, serum albumin; SAIL, Secure Anonymised Information Linkage.

### Trends in serum blood testing over time

The percentage of patients receiving an ALT test in each year rose from 2.10% (95% CI 2.08% to 2.12%) in 2000 to 32.55% (95% CI 32.49% to 32.61%) in 2017. In the same period, AST testing decreased from 2.67% (95% CI 2.64% to 2.70%) to 1.06% (95% CI 1.05% to 1.08%) (figure 1, online supplemental table S4). The same pattern shown in figure 1 was also found separately for males and females, with males receiving fewer AST and in particular ALT tests each year (around 3% less in 2005 to 6% less by 2017) than females (online supplemental figure S5). This trend is echoed across the whole study period in general with around 9% more females receiving an ALT compared with males (table 1).

The measurement of serum albumin increased sharply between 2000 and 2004 from 4% to 21% and then rose more slowly to 33% by 2017. Platelet count measurement followed a similar trend, rising from 7% to 21% in 2004 with a more gradual increase to 33% by 2017 (figure 1). Serum albumin and platelet testing was higher in females across the study (online supplemental figure S6).

### Trends in blood testing in different age groups over time

Figure 2 illustrates the percentage of SAIL patients tested with at least one liver blood test in each age band for every year of the study and shows there was an increase in the proportion of patients being tested with age. Those least likely to receive at least one liver blood test were in the 18–39 age category with patients in the 80+age band most likely to be tested. The time trends stratified by age for each individual marker are shown in online supplemental figures S7–S10. These all have a similar pattern to the overall trends for any marker in figure 2.

### Distribution of ALT and AST values by age and sex

Figure 3 and online supplemental table S5 illustrate how the values of ALT and AST depend both on sex and the age of the subject. This is especially true of the ALT in male subjects where the upper range of values considered normal as denoted by the 95th percentile, has a maximum at age 30 of 90 U/L which rapidly decreases with age to 45 U/L by age 80. The difference was less marked in females, where the ALT 95th percentile reached a maximum of 55 U/L at the older age of 50, and then dropped to under 40 U/L by age 80. AST was also age dependent for men and women, but not to the same degree as ALT.

## Discussion

### Main findings

We have found that in Wales the routine use of ALT has increased dramatically since 2000 rising from 2% to 33%. At the same time the use of the AST test has reduced from 3% to 1%. Importantly, we noted that there were large differences in the population distribution (ranges) of ALT with increasing age in men and women, although the differences were most marked for men. This altered distribution was also true for AST with men, although less prominent for women.

### Strengths of our approach

This work uses a very large, unselected cohort taken from a complete nation within the UK population which includes the full range of age groups and patient presentations to their GPs. The test results we have used have come from blood samples that would have been analysed by a variety of different laboratories and so represent the real-world variation observed in clinical practice. In addition, the full range of liver disease aetiologies will be included, all with different levels of fibrosis from mild to cirrhosis. In addition, less than 5% of eligible patients were excluded from our cohort (which is representative of the Welsh population) due to missing, duplicated or incorrect data (online supplemental figure S1). This means we can make representative statements about the overall state of liver blood testing in the population as a whole and will enable our future work looking into identifying cases of chronic liver disease earlier to be applicable to the whole spectrum of liver disease.

We have concentrated on the indirect serum markers rather than the direct markers, as the latter are more expensive to carry out and can be influenced by other inflammatory processes, such as rheumatic disorders, and may not detect extensive fibrosis when minimal inflammation is present.^6^

### Limitations

The main limitation of our study is that it relies on opportunistic blood test measurements taken for other indications. Therefore, their values will not reflect the true population distribution of the blood test results and is likely to be biased towards abnormal values. This may not be ideal as we are attempting to define upper limits of normal (ULN) values using percentiles, which would usually be generated from a healthy population. However, universal blood testing in a population is expensive and is not feasible outside of expensive cohorts. Therefore, our study reflects the distribution of blood test results in patients accessing healthcare, and the reality in implementing any opportunistic screening.

To examine whether our results are overly biased towards abnormal test results from patients with poorer health status, rather than healthy individuals, we conducted a sensitivity analysis and repeated the calculations of median blood test results shown in table 1 and online supplemental table S5, using only one random value from each patient. The results shown in online supplemental table S6 illustrate this makes very little difference to the results, with the overall pattern of values by age and sex also being preserved.

Although by 2017 around 68% of those aged 80 and over, and around 58% of those aged 60–79 were tested, the overall percentage of the SAIL population (which represents 79% of the Welsh population) receiving a relevant serum blood test is still quite low at 38% (figure 2). So, in using opportunistic liver screening from these blood tests, a large proportion of the populace would be omitted. Further work would need to be done to ascertain if those who go on to develop liver disease are also those who would have had blood tests performed, or whether they would have been missed and more widespread liver screening would be needed to identify these cases.

This study has not considered comorbidities and other factors that may influence the levels of the enzymes we are investigating or be an indication to do the test. For example, ALT and AST are influenced by diabetes, obesity, cardiovascular disease, hepatitis and certain medications such as paracetamol and statins. We could not show trends by different comorbidities as these were not available in the SAIL denominator data.

### Comparison with the literature

The need for earlier and more accurate identification of liver disease has become more important due to its rapid increase in recent years. For example, a recent health policy report for the Lancet, showed the number of hospital admissions related to liver disease was still increasing in the UK, in England in particular.^20 21^ The 2014 Lancet Commission report also found that increases in alcohol-related liver disease (ALD) and NAFLD have paralleled rising levels of alcohol use and obesity, respectively, with three-quarters of deaths from liver disease attributed to excess alcohol consumption. Across all age groups, mortality rates from liver disease in the UK have increased by 400% since 1970. The figure is even higher in patients under 65 years. Although ALD and NAFLD are the main causes, chronic viral hepatitis B and C also play a part. However, the opposite has happened in some other Western European countries, such as France, who have implemented controls on alcohol advertising and widespread use of simple non-invasive diagnostic techniques for early detection.^22^

The reasons for the increased rate of ALT testing with the reverse trend for AST in recent years is unclear, but we can speculate. The recently updated British Society of Gastroenterology guidelines,^23^ although not applicable to screening, do omit the AST in the panel of tests for initial investigation of specific, advanced liver diseases. However, these guidelines were produced in 2018, after the end of our study, so their implementation cannot be directly linked in a causal way to our findings. A review from over 20 years ago which precedes our study time period, although not recommending it for diagnosing disease severity, put forward the ALT as a means of screening for acute and chronic liver disease in certain high-risk populations, without the need for also measuring the AST.^24^

ALT level might be preferred over AST in the acute assessment of at risk patients, as it is more liver specific as a more accurate guide to liver inflammation than AST level, which can be affected by ongoing processes in other organs besides the liver.^25^

Another possible reason for the reduction in AST testing could be due to cost-saving measures. Each additional test carried out costs money, and if it were felt the ALT was sufficient for an initial liver investigation, then the AST would be surplus to requirements. For example, a Swedish study from 1999,^26^ which implemented an education programme in primary care, showed that the use of the AST in primary care could be reduced and resulted in significant savings in healthcare costs, and as a result its use fell by 67% in the county of Uppsala in Sweden. A US study^27^ recommended that roughly US$100 million could be saved annually if the AST was only carried out in tandem with the ALT when the latter exceeded a predetermined limit (35 U/L), and that only a small proportion (0.5%–2.0%) of outpatients with abnormally high liver enzymes would be missed as a result. A similar result was found in a Canadian study.^28^ The AST still has value in certain settings however, and there is still no consensus on the optimal use of this test.^29^

Finally, our finding of the dependence between reference limits for ALT and AST and age and sex, confirm that of a recent German study,^30^ with a similar population to ours (in terms of age range and including all test data regardless of indication). In this study they analysed over 1.3 million blood samples taken from primary and secondary care locations in northern Germany. The relationship between ALT and age also persisted in a routine health-check subpopulation and to a lesser extent, a healthy subpopulation of their cohort. This phenomenon has been highlighted previously,^31 32^ but 20 years later has still not been incorporated into guidelines or clinical practice. The findings of our study show that the 95th percentile exceeded values of 90 U/L at age 30 and fell to below 50 U/L by age 80. Therefore, the proportion of men categorised as having an elevated ALT is different for different age groups as currently the same ULN is applied at each age.

Although the relationship between ALT and age and sex we have demonstrated in the Welsh population would probably also exist more generally in the whole UK population, and it has also been found in a German study, this phenomenon requires investigation in further populations such as those in Asia and other settings worldwide.

The reasons for the reduction in ALT level with age are unclear. It is well established that there are structural changes in the liver as we age, including a reduction in the liver’s overall volume.^33^ It has been shown that the reduction in ALT production with age is not due to factors associated with muscle mass, such as BMI and hip-to-waist ratio, as ALT seems to be independent of these.^34^ It has also been shown that as ALT reduces with age, although not being associated with lean body mass, it is highly correlated with AST level.^35^ AST might be expected to be influenced more by ageing and therefore muscle mass, as it is less liver specific than ALT and occurs in many more locations in the body. However, we have shown that AST by comparison does not depend so highly on age as does ALT. These facts suggest the reduction in ALT and AST with time is a normal part of the ageing process and related more to liver ageing than anything else.

This has implications for the straightforward interpretation of these blood tests and the construction of fibrosis markers like the AST/ALT ratio, where the ULN or reference value, for ALT and AST may need to be altered depending on the age and sex of the patient.

## Conclusions

Despite the rise in liver disease in recent years, the routine use of AST in the Welsh population has at the same time decreased, possibly due to official guidelines. This is in contrast to the overall rise in venous blood testing as shown by the rapid increase in the use of the other blood markers. The reduction in AST testing is concerning as many composite liver fibrosis markers depend on it. Therefore, to implement opportunistic, widespread screening for liver disease will require either a reversal of this trend and the reinstatement of the AST in the standard liver blood panel test, or new algorithms not dependent on the AST, such as the recently reported CIRRhosis Using Standard tests,^36^ will need developing.

We have also shown important differences between age groups and sexes in what would be considered an abnormal ALT (and to a lesser extent AST). Adapting fibrosis markers to include adjustments for these age and sex differences is likely to be of crucial importance in future.

## Data Availability

Data may be obtained from a third party and are not publicly available. The data used in this study are available in the SAIL databank at Swansea University, Swansea, UK. All proposals to use SAIL data are subject to review by an independent Information Governance Review Panel (IGRP). Before any data can be accessed, approval must be given by the IGRP. The IGRP gives careful consideration to each project to ensure proper and appropriate use of SAIL data. When access has been granted, it is gained through a privacy-protecting safe haven and remote access system referred to as the SAIL Gateway. SAIL has established an application process to be followed by anyone who would like to access data via SAIL https://www.saildatabank.com/application-process.
