# Supplementary material for: Trends in indirect liver function marker testing in Wales from 2000 to 2017 and their association with age and sex: an observational study
Source: BMJ Open Gastroenterol. 2022 Apr 29;9(1):e000885. doi: 10.1136/bmjgast-2022-000885 (PMC9058682; doi:10.1136/bmjgast-2022-000885)
Supplement: Supplementary data [file bmjgast-2022-000885supp001.pdf]

Supplemental material
TH, 21/03/2022

Trends in indirect liver function marker testing in Wales from 2000 to 2017 and their association with age and sex

List of tables and figures:

Tables

Table S1: Liver function test Read codes included in the data, by type

Table S2: Frequency and percentage of blood tests in each month, all years (2000-2017) combined

Table S3: The number of patients tested in each year of the study, by sex

Table S4: Counts and percentages of patients receiving each blood serum test in each year with 95% CIs

Table S5: Median (IQR) blood serum results by age band and sex

Table S6: Median (IQR) blood serum results by age band and sex, random sample

Figures

Figure S1: Summary of data cleaning steps applied to the LFT data

Figure S2: Percentage of blood tests in each month, for all years (2000 to 2017) combined

Figure S3: Percentage of patients tested who are male and female in each year of the study.

Figure S4: Distribution of the final test values for each of the four serum tests

Figure S5: Percentage of SAIL patients receiving an AST or ALT test for each year of the study, by sex.

Figure S6: Percentage of SAIL patients receiving a platelet count or serum albumin test for each year of the study, by sex.

Figure S7: Percentage of SAIL patients in each age group receiving at least one ALT test for each year of the study.

Figure S8: Percentage of SAIL patients in each age group receiving at least one AST test for each year of the study.

Figure S9: Percentage of SAIL patients in each age group receiving at least one serum albumin test for each year of the study.

Figure S10: Percentage of SAIL patients in each age group receiving at least one platelet count test for each year of the study.

Table S1: Liver function test Read codes included in the data, by type

Blood test type	Read code and description
Platelet count	42P.. Platelet count<br>42P1. Platelet count normal<br>42P4. Platelet count abnormal<br>42PZ. Platelet count NOS
Serum albumin	44M4. Serum albumin<br>44M40 Serum albumin normal<br>44M41 Serum albumin low<br>44MI. Plasma albumin level
ALT	44G3. ALT/SGPT serum level<br>44G30 ALT/SGPT level normal<br>44G31 ALT/SGPT level abnormal<br>44GA. Plasma alanine aminotransferase level<br>44GB. Serum alanine aminotransferase level<br>44S8. Serum alanine level
AST	44H5. AST – aspartate transam. (SGOT)<br>44H50 AST/SGOT level normal<br>44H51 AST/SGOT level abnormal<br>44H52 AST/SGOT level raised<br>44HB. AST serum level<br>44HC. Plasma aspartate transaminase level

Table S2: Frequency and percentage of blood tests in each month, all years (2000-2017) combined

Month	Frequency	Percentage
January	3,386,336	8.45
February	3,276,984	8.18
March	3,544,346	8.84
April	3,212,457	8.02
May	3,293,831	8.22
June	3,417,312	8.53
July	3,462,340	8.64
August	3,219,211	8.03
September	3,465,670	8.65
October	3,576,356	8.92
November	3,464,108	8.64
December	2,758,734	6.88
Total	40,077,685	100.00

Treating each individual serum result (ALT, AST, serum albumin and platelet count) as a separate test.

Table S3: The number of patients tested in each year of the study, by sex

Year	No. males	Perc. males	No. females	Perc. females	Total
2000	45,186	37.12	76,542	62.88	121,728
2001	62,740	37.98	102,457	62.02	165,197
2002	90,595	39.23	140,362	60.77	230,957
2003	140,839	40.30	208,669	59.70	349,508
2004	211,342	41.21	301,505	58.79	512,847
2005	238,534	41.68	333,709	58.32	572,243
2006	257,580	42.17	353,205	57.83	610,785
2007	273,338	42.66	367,412	57.34	640,750
2008	284,638	42.73	381,531	57.27	666,169
2009	293,114	42.84	391,020	57.16	684,134
2010	299,552	43.03	396,664	56.97	696,216
2011	312,238	42.99	414,021	57.01	726,259
2012	321,432	43.31	420,731	56.69	742,163
2013	322,807	43.26	423,337	56.74	746,144
2014	325,391	43.01	431,090	56.99	756,481
2015	328,123	42.95	435,843	57.05	763,966
2016	335,907	42.93	446,505	57.07	782,412
2017	340,290	42.88	453,318	57.12	793,608

Each patient is counted only once in each year, even if they have multiple blood tests, but may be counted in more than one year. Therefore, the sum of the totals for all years is greater than the total number of individual patients in the study. Percentages are row percentages and with respect to the cohort, not the whole SAIL population.

Table S4: Counts and percentages of patients receiving each blood serum test in each year with 95% CIs

		AST		ALT		Serum albumin		Platelet count	
**Year**	**Total SAIL patients**	**No. patients**	**Percentage of SAIL patients (95% CI)[1]**	**No. patients**	**Percentage of SAIL patients (95% CI)[1]**	**No. patients**	**Percentage of SAIL patients (95% CI)[1]**	**No. patients**	**Percentage of SAIL patients (95% CI)[1]**
2000	1,484,723	39,627	2.67 (2.64 – 2.70)	31,159	2.10 (2.08 – 2.12)	62,176	4.19 (4.16 – 4.22)	101,041	6.81 (6.76 – 6.85)
2001	1,601,810	62,015	3.87 (3.84 – 3.90)	44,449	2.77 (2.75 – 2.80)	93,244	5.82 (5.78 – 5.86)	135,058	8.43 (8.39 – 8.47)
2002	1,773,563	89,293	5.03 (5.00 – 5.07)	79,338	4.47 (4.44 – 4.50)	144,035	8.12 (8.08 – 8.16)	182,074	10.27 (10.22 – 10.31)
2003	1,823,387	129,178	7.08 (7.05 – 7.12)	154,605	8.48 (8.44 – 8.52)	249,354	13.68 (13.63 – 13.73)	265,124	14.54 (14.49 – 14.59)
2004	1,871,818	215,844	11.53 (11.49 – 11.58)	220,711	11.79 (11.75 – 11.84)	401,616	21.46 (21.40 – 21.51)	387,327	20.69 (20.63 – 20.75)
2005	1,901,078	184,093	9.68 (9.64 – 9.73)	331,771	17.45 (17.40 – 17.51)	463,725	24.39 (24.33 – 24.45)	430,271	22.63 (22.57 – 22.69)
2006	1,921,500	190,664	9.92 (9.88 – 9.97)	365,031	19.00 (18.94 – 19.05)	504,655	26.26 (26.20 – 26.33)	457,302	23.80 (23.74 – 23.86)
2007	1,946,078	201,607	10.36 (10.32 – 10.40)	383,427	19.70 (19.65 – 19.76)	535,924	27.54 (27.48 – 27.60)	488,686	25.11 (25.05 – 25.17)
2008	1,957,636	211,132	10.79 (10.74 – 10.83)	400,798	20.47 (20.42 – 20.53)	558,174	28.51 (28.45 – 28.58)	518,712	26.50 (26.44 – 26.56)
2009	1,969,443	149,507	7.59 (7.55 – 7.63)	456,369	23.17 (23.11 – 23.23)	575,185	29.21 (29.14 – 29.27)	543,414	27.59 (27.53 – 27.65)
2010	1,990,739	108,205	5.44 (5.40 – 5.47)	484,956	24.36 (24.30 – 24.42)	587,897	29.53 (29.47 – 29.60)	563,735	28.32 (28.26 – 28.38)
2011	2,006,560	48,265	2.41 (2.38 – 2.43)	513,110	25.57 (25.51 – 25.63)	618,872	30.84 (30.78 – 30.91)	597,403	29.77 (29.71 – 29.84)
2012	2,018,864	10,567	0.52 (0.51 – 0.53)	527,291	26.12 (26.06 – 26.18)	634,635	31.44 (31.37 – 31.50)	620,420	30.73 (30.67 – 30.79)
2013	2,030,414	10,729	0.53 (0.52 – 0.54)	546,483	26.91 (26.85 – 26.98)	641,313	31.59 (31.52 – 31.65)	627,749	30.92 (30.85 – 30.98)
2014	2,048,661	10,179	0.50 (0.49 – 0.51)	582,185	28.42 (28.36 – 28.48)	656,455	32.04 (31.98 – 32.11)	647,124	31.59 (31.52 – 31.65)
2015	2,072,213	10,641	0.51 (0.50 – 0.52)	651,910	31.46 (31.40 – 31.52)	667,941	32.23 (32.17 – 32.30)	664,705	32.08 (32.01 – 32.14)
2016	2,095,687	14,440	0.69 (0.68 – 0.70)	675,027	32.21 (32.15 – 32.27)	689,808	32.92 (32.85 – 32.98)	686,715	32.77 (32.70 – 32.83)
2017	2,115,961	22,469	1.06 (1.05 – 1.08)	688,756	32.55 (32.49 – 32.61)	703,248	33.24 (33.17 – 33.30)	703,509	33.25 (33.18 – 33.31)

These percentages are also shown graphically in Figure 1 in the main paper.

[1] 95% CI calculated using the Exact method in Stata/MP 17.0 using the *cii proportions* command.

Table S5: Median (IQR)[1] blood serum results by age band and sex

	**All patients**	**Males**	**Females**
Number of patients[2]	2,145,178 (100.00%)	951,926 (44.38%)	1,193,252 (55.62%)
Platelet count (10^9/L)			
18-39	263 (224 – 310)	248 (212 – 289)	269 (229 – 316)
40-59	266 (224 – 314)	247 (209 – 290)	279 (237 – 327)
60-79	254 (210 – 305)	234 (194 – 281)	270 (227 – 321)
80+	249 (202 – 305)	224 (182 – 275)	264 (217 – 320)
Serum albumin (g/L)			
18-39	44 (41 – 46)	45 (42 – 48)	43 (40 – 46)
40-59	43 (40 – 45)	43 (41 – 46)	42 (39 – 45)
60-79	42 (39 – 44)	42 (39 – 45)	42 (39 – 44)
80+	40 (37 – 43)	40 (37 – 43)	40 (37 – 43)
ALT (U/L)			
18-39	21 (14 – 33)	30 (20 – 46)	17 (13 – 25)
40-59	25 (17 – 36)	30 (22 – 43)	20 (15 – 29)
60-79	21 (16 – 29)	23 (17 – 32)	19 (15 – 27)
80+	16 (12 – 21)	17 (13 – 23)	15 (12 – 20)
AST (U/L)			
18-39	21 (17 – 27)	25 (20 – 33)	19 (16 – 23)
40-59	23 (19 – 29)	25 (21 – 32)	21 (17 – 26)
60-79	23 (19 – 28)	24 (20 – 29)	22 (18 – 27)
80+	22 (18 – 26)	22 (18 – 27)	21 (18 – 26)

[1] Includes all blood test data, so some patients are included more than once in each age band and/or in multiple age bands. [2] The percentage is with respect to the cohort.

Table S6: Median (IQR) blood serum results by age band and sex, random sample[1]

	All patients	Males	Females
All ages combined			
Platelet count (10^9/L)	258 (218 – 305)	242 (205 – 285)	271 (230 – 318)
Serum albumin (g/L)	43 (40 – 45)	43 (40 – 46)	42 (39 – 45)
ALT (U/L)	20 (15 – 30)	25 (18 – 35)	18 (13 – 25)
AST (U/L)	22 (18 – 27)	24 (20 – 30)	20 (17 – 25)
Platelet count (10^9/L)			
18-39	260 (222 – 304)	246 (212 – 286)	269 (230 – 314)
40-59	262 (223 – 307)	247 (211 – 288)	276 (236 – 321)
60-79	254 (213 – 302)	236 (199 – 281)	270 (229 – 318)
80+	250 (205 – 307)	228 (187 – 279)	265 (219 – 322)
Serum albumin (g/L)			
18-39	44 (41 – 47)	45 (42 – 48)	43 (40 – 46)
40-59	43 (40 – 45)	44 (41 – 46)	43 (40 – 45)
60-79	42 (39 – 44)	42 (39 – 45)	42 (39 – 44)
80+	40 (36 – 43)	40 (36 – 43)	40 (36 – 43)
ALT (U/L)			
18-39	20 (14 – 31)	28 (19 – 41)	17 (13 – 23)
40-59	24 (17 – 34)	29 (21 – 40)	19 (15 – 27)
60-79	21 (16 – 28)	23 (17 – 31)	19 (15 – 25)
80+	16 (12 – 21)	17 (13 – 23)	15 (11 – 20)
AST (U/L)			
18-39	21 (17 – 26)	24 (20 – 31)	19 (16 – 23)
40-59	22 (18 – 28)	25 (21 – 31)	20 (17 – 25)
60-79	23 (19 – 28)	23 (19 – 29)	22 (18 – 26)
80+	22 (18 – 26)	22 (18 – 27)	21 (18 – 26)

[1] One random test per patient over the whole study period for all ages combined, and one random test per patient, per age band, for medians within each age band.

NB: the number of patients in each category varies due to not all patients receiving all tests and not being tested at all ages. The numbers range from 846,946 patients in the 40-59 age band receiving a platelet count test to 31,626 males in the 80+ age band receiving an AST test.

Figure S1: Summary of data cleaning steps applied to the LFT data

Box 1: All available liver function test data

Type	Frequency	Percentage
Platelet count	16,289,931	26.91
Serum albumin	17,399,272	28.74
ALT	12,677,940	20.95
AST	3,119,736	5.15
Liver function tests	9,797,468	16.19
Liver enzyme tests	1,245,684	2.06
**Total**	60,530,031	100.00

Initial number of patients
N = 2,408,176

Final number of patients
N=2,145,178 (89.1%)

Box 2: Patients opted out of research
N=117 patients dropped (1,763 obs).

Box 3: Patients with data outside the 2000 to 2017 year range, with missing dates, and duplicate observations.
N=100,952 patients dropped (3,311,360 observations in total).

Box 4: Patients with liver function and liver enzyme test data only that could not be assigned to any specific test type.
N=13,625 patients dropped (10,520,920 observations in total).

Box 5: Patients with test result values that were missing, unlikely, non-integer or recorded in unknown units.
N=4,000 patients dropped (590,772 observations in total).

Box 6: Patients with duplicated test results on the same day.
No patients dropped (5,133,829 observations dropped).

Box 7: Patients with three repeat tests on the same day or two tests where the difference between the results is greater than the global IQR for all the data of the same test type.
N=115 patients dropped (41,802 observations in total).

Box 8: Patients with inconsistent sex or date of birth data and/or assigned the same local patient ID as another patient.
N=8,569 patients dropped (141,835 observations in total).

Box 9: Tested only before aged 18 years.
N=135,620 patients dropped
(710,065 observations in total).

Box 10: Final data (aged 18+ at time of test)

Type	Frequency	Percentage
Platelet count	13,638,831	34.03
Serum albumin	13,271,457	33.11
ALT	10,656,162	26.59
AST	2,511,235	6.27
**Total**	40,077,685	100.00

Total number of patients dropped
n = 262,998 (10.9%)

Total number of tests dropped
n= 20,452,346 (33.8%)

Figure S2: Percentage of blood tests in each month, for all years (2000 to 2017) combined

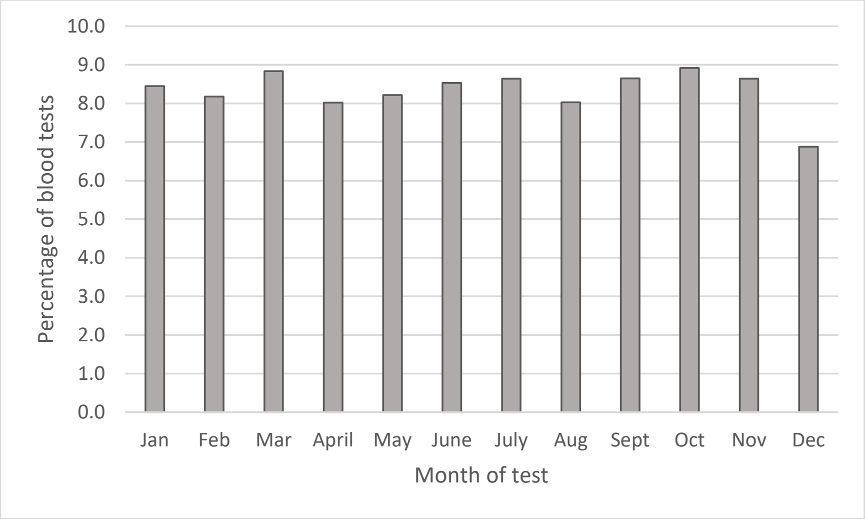

Note that individual test results are being counted here, not venous samples. Percentages are for all years combined, although individual years show very similar patterns across the months.

Figure S3: Percentage of patients tested who are male and female in each year of the study

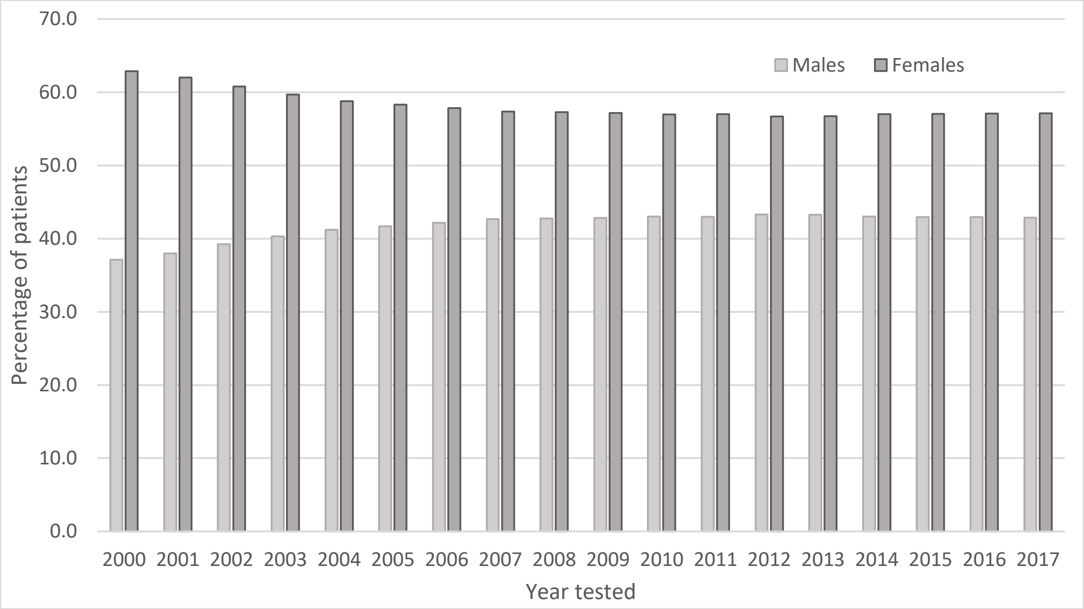

Patients are counted once in each year, even if they have several blood tests, but may be counted in more than one year. The percentage is with respect to the cohort, not the whole SAIL population.

Figure S4: Distribution of the final test values for each of the four serum tests

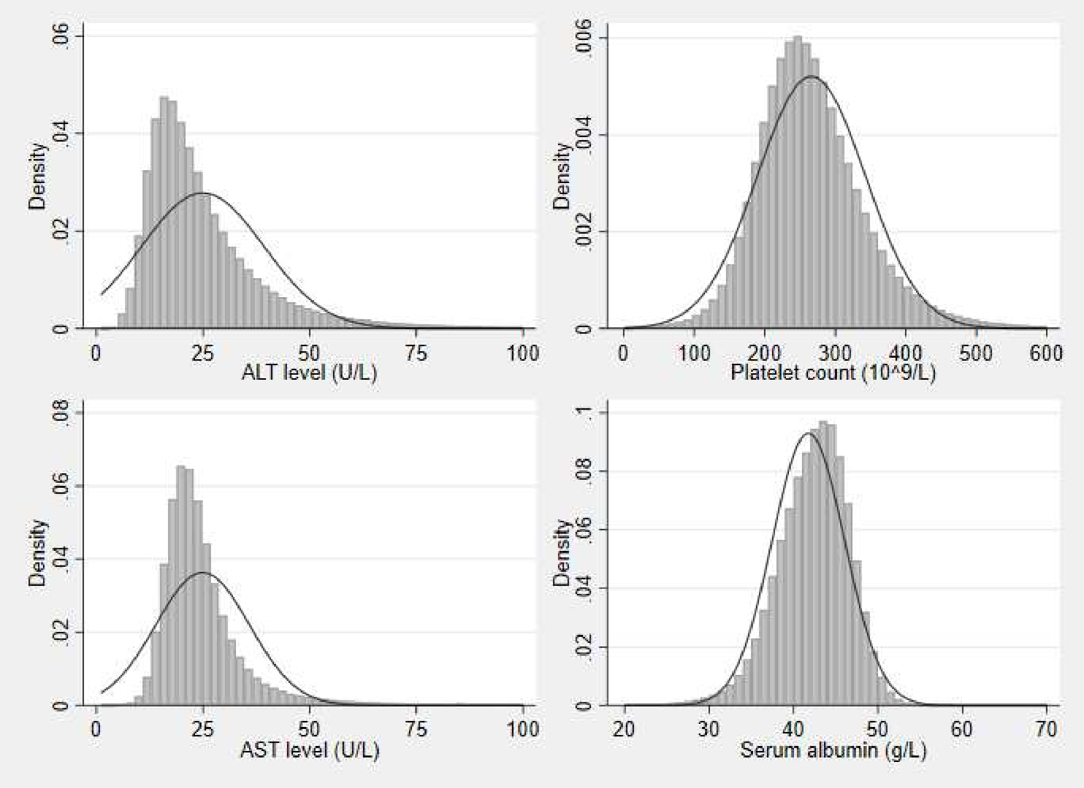

Each plot includes data from all years, 2000 to 2017, however note that data in the extreme tails of the distributions is not shown. The plot of ALT includes 10,523,328 data values (n=132,834, 1.25% values above 100 not shown), the plot of AST includes 2,483,218 data values (n=28,017, 1.12% values above 100 not shown), the plot of platelet count includes 13,574,676 data values (n=64,155, 0.47% values above 600 not shown), and the plot of serum albumin includes 13,263,302 data values (n=8,155, 0.06% values outside the 20-70 range not shown). Platelet count and serum albumin have normal distributions, shown by the superimposed line, but ALT and AST are both right-skewed and both feature a high peak in the middle.

Figure S5: Percentage[1] of SAIL patients receiving an AST or ALT test for each year of the study, by sex.

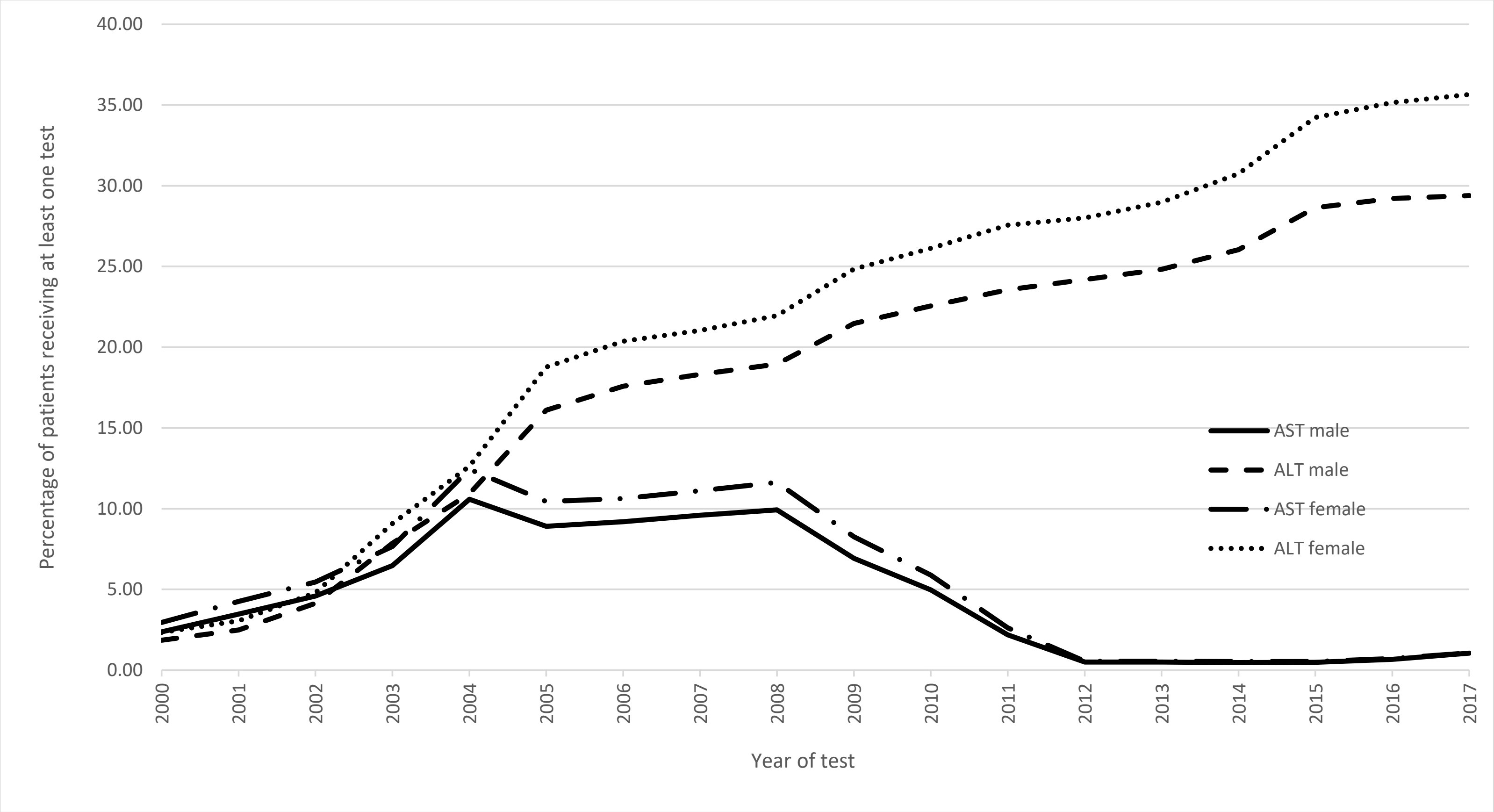

[1] The percentages for each year are generated as follows: The denominator is the total number of SAIL patients of the same sex in that year for whom GP data was available. The numerator is the total number of study participants of that sex, in that year who had that particular test. The figure is interpreted as follows: in 2008, 10% of male SAIL patients received an AST test, whereas 12% of female SAIL patients in the same year received the AST test.

Figure S6: Percentage[1] of SAIL patients receiving a platelet count or serum albumin test for each year of the study, by sex.

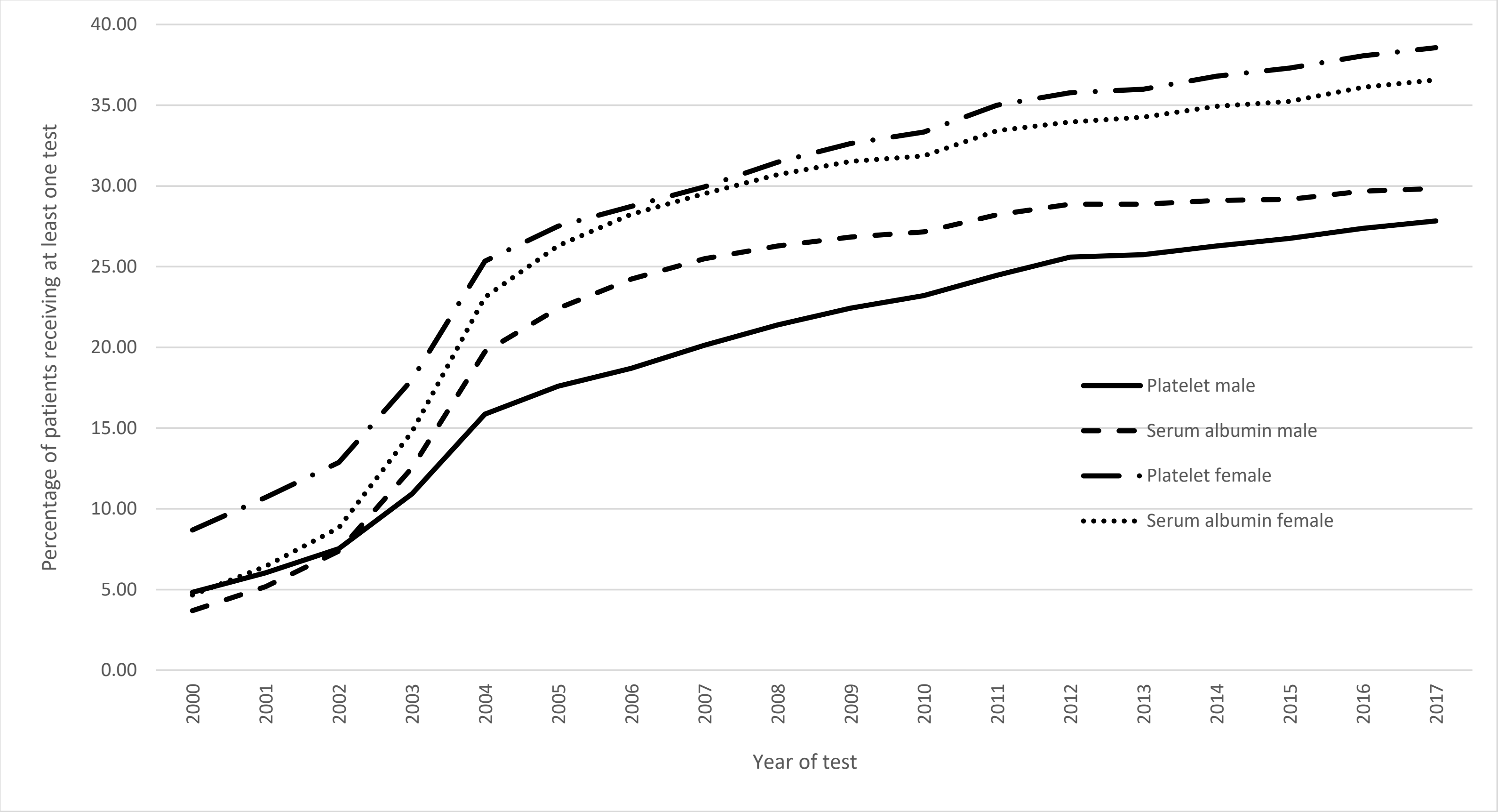

[1] The percentages for each year are generated as follows: The denominator is the total number of SAIL patients of the same sex in that year for whom GP data was available. The numerator is the total number of study participants of that sex, in that year who had that particular test. The figure is interpreted as follows: in 2012, 26% of male SAIL patients received a platelet count test, whereas 36% of female SAIL patients in the same year received a platelet count test.

Figure S7: Percentage[1] of SAIL patients in each age group receiving at least one ALT test for each year of the study.

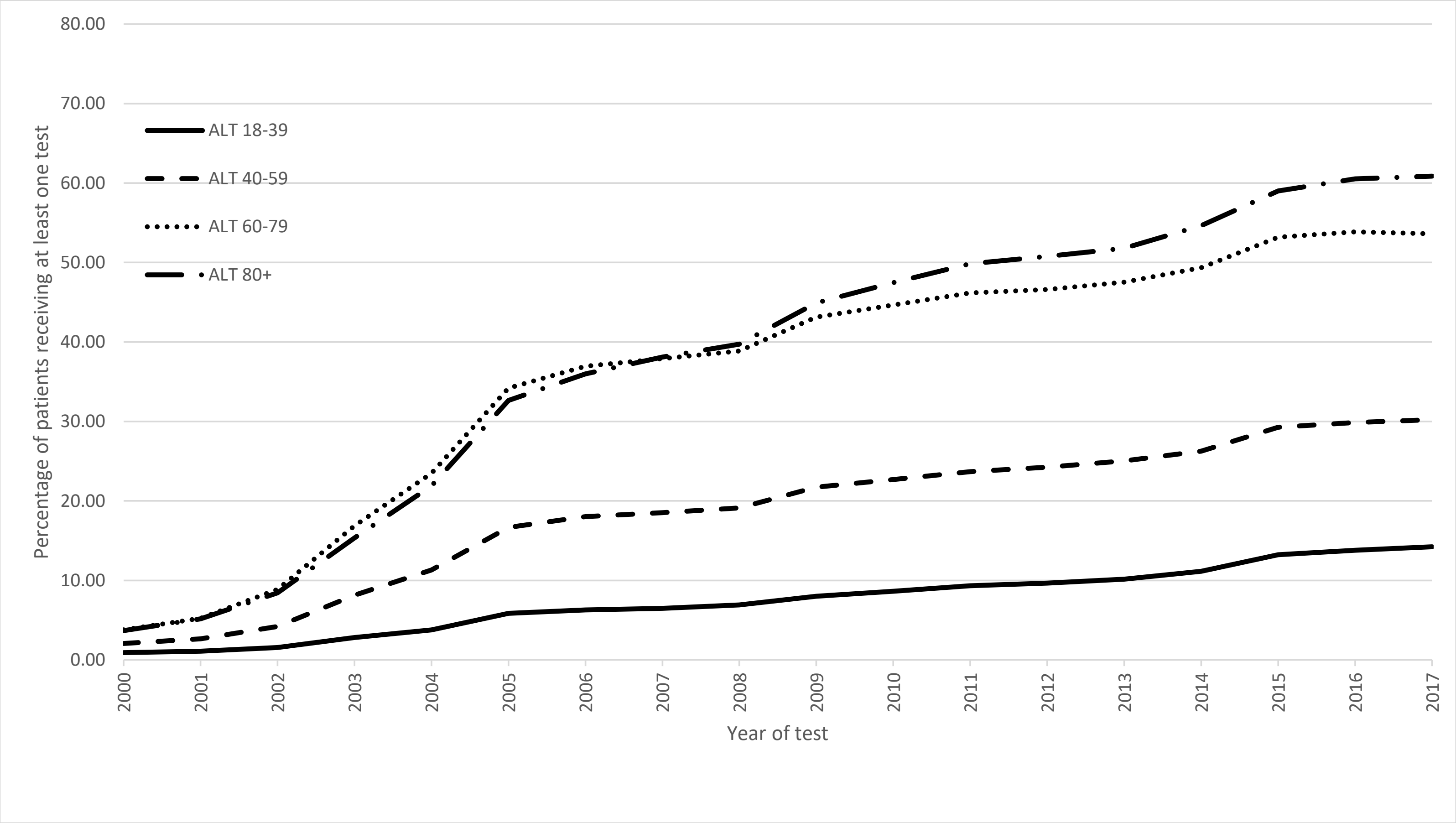

[1] The percentages for each year are generated as follows: The denominator is the total number of SAIL patients in that age group and year for whom GP data was available. The numerator is the total number of study participants in that age group and year who had an ALT test. The figure is interpreted as follows: in 2016, 30% of SAIL patients aged 40-59 received an ALT test, whereas 60% of SAIL patients aged 80+ received the test.

Figure S8: Percentage[1] of SAIL patients in each age group receiving at least one AST test for each year of the study.

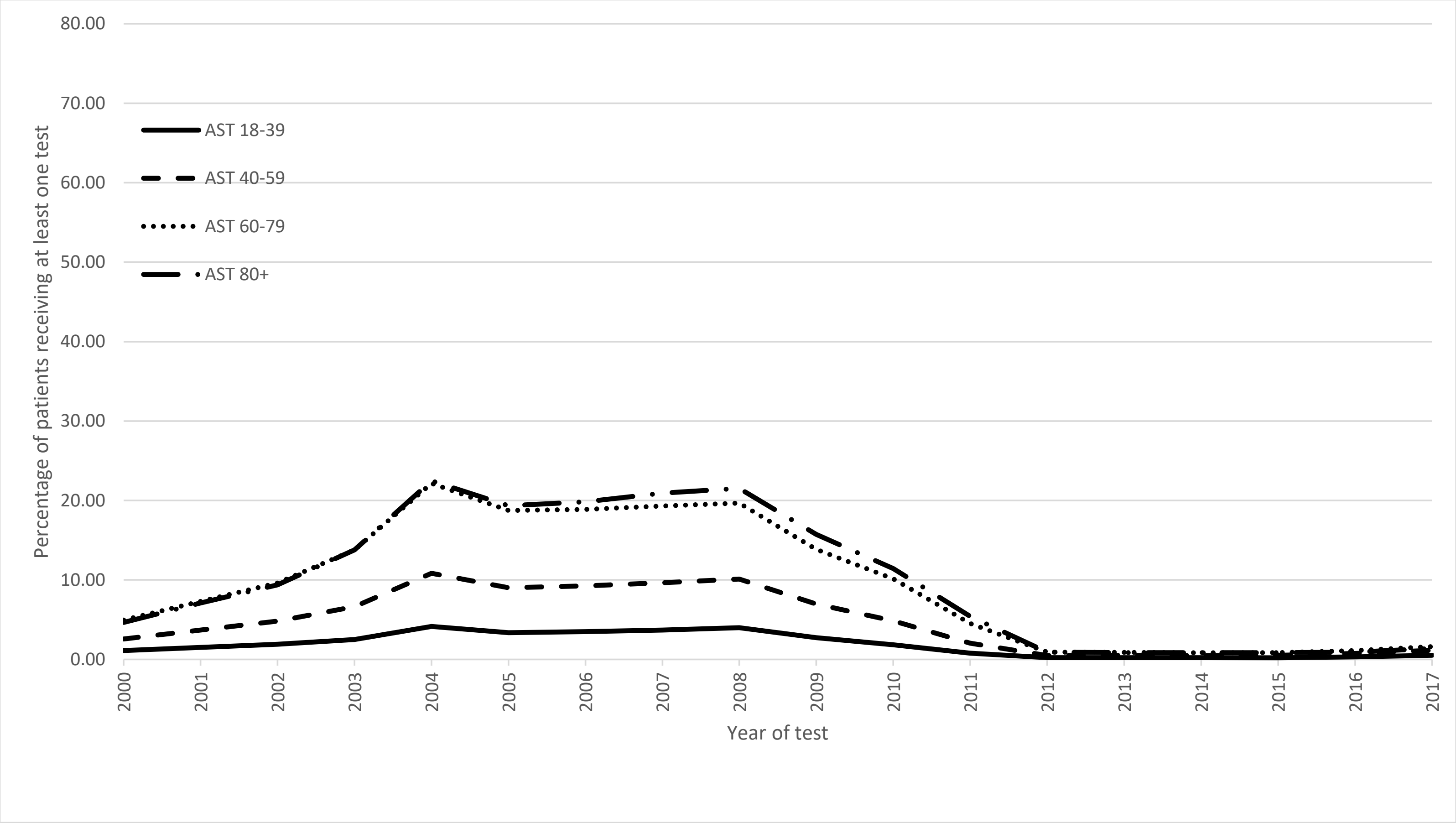

[1] The percentages for each year are generated as follows: The denominator is the total number of SAIL patients in that age group and year for whom GP data was available. The numerator is the total number of study participants in that age group and year who had an AST test. The figure is interpreted as follows: in 2004, 4% of SAIL patients aged 18-39 received an AST test, whereas 11% of SAIL patients aged 40-59 received the test.

Figure S9: Percentage[1] of SAIL patients in each age group receiving at least one serum albumin test for each year of the study.

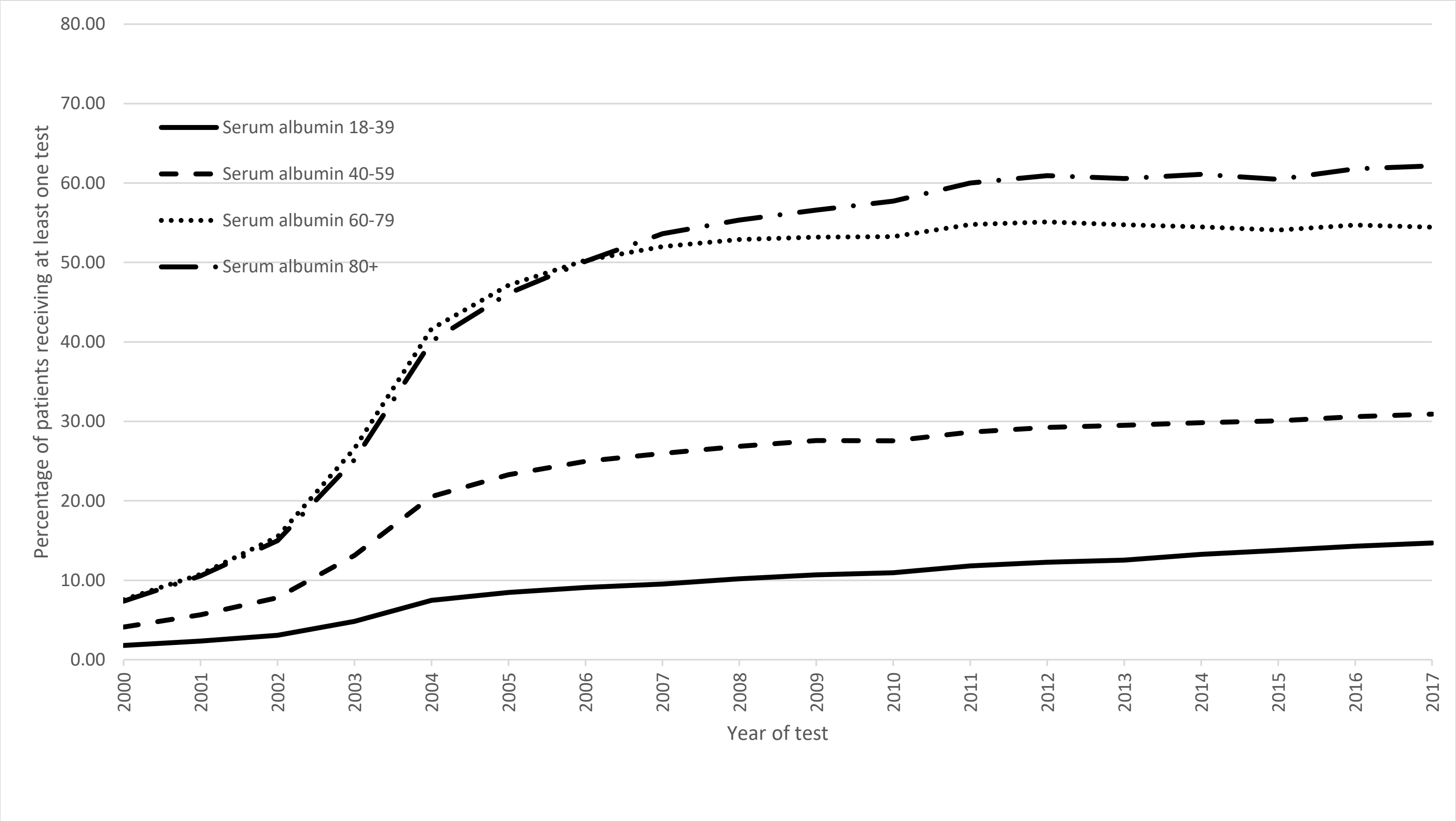

[1] The percentages for each year are generated as follows: The denominator is the total number of SAIL patients in that age group and year for whom GP data was available. The numerator is the total number of study participants in that age group and year who had a serum albumin test. The figure is interpreted as follows: in 2008, 10% of SAIL patients aged 18-39 received a serum albumin test, whereas 27% of SAIL patients aged 40-59 received the test.

BMJ Publishing Group Limited (BMJ) disclaims all liability and responsibility arising from any reliance placed on this supplemental material which has been supplied by the author(s)

Figure S10: Percentage[1] of SAIL patients in each age group receiving at least one platelet count test for each year of the study.

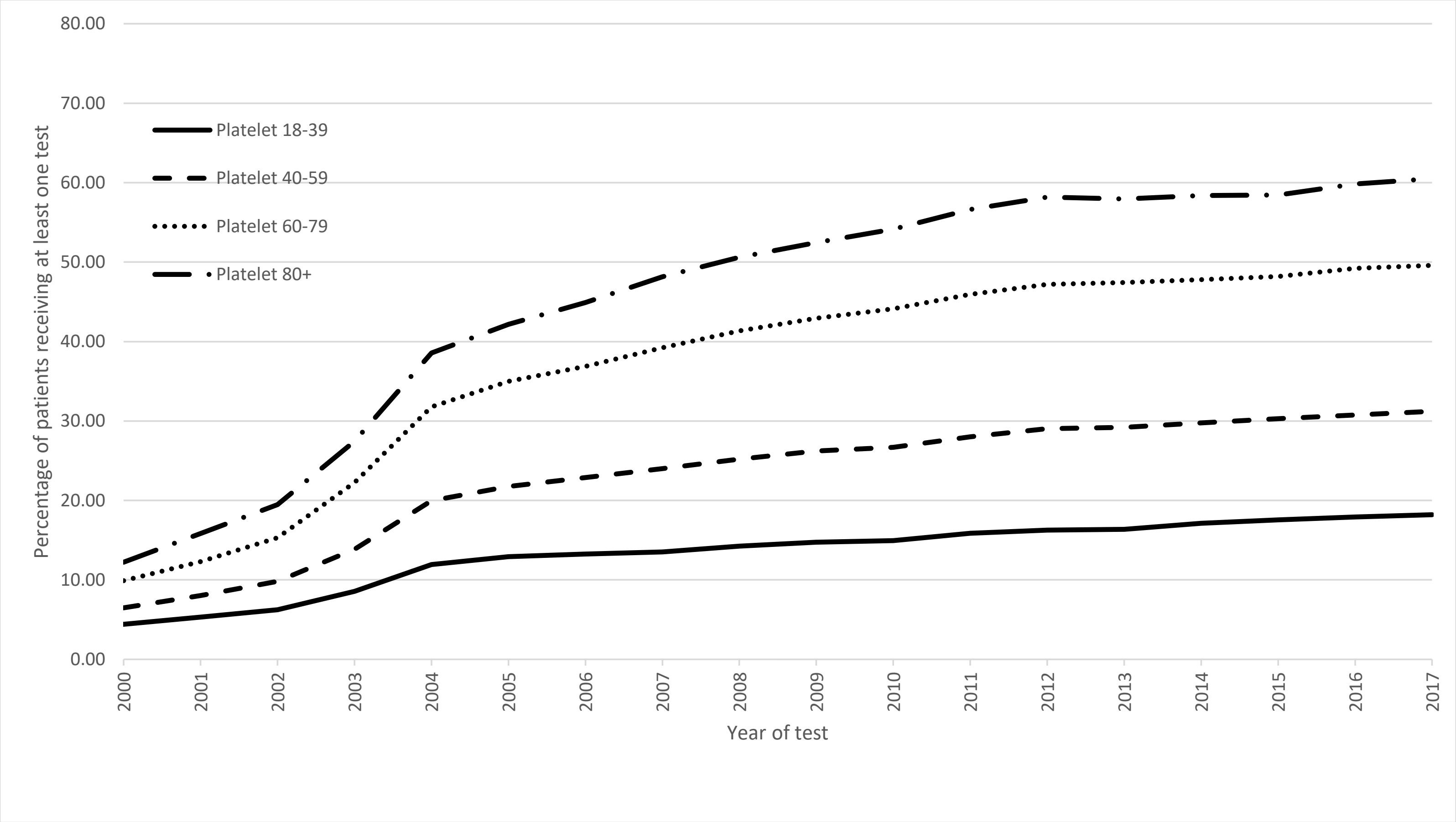

[1] The percentages for each year are generated as follows: The denominator is the total number of SAIL patients in that age group and year for whom GP data was available. The numerator is the total number of study participants in that age group and year who had a platelet count test. The figure is interpreted as follows: in 2010, 15% of SAIL patients aged 18-39 received a platelet count test, whereas 44% of SAIL patients aged 60-79 received the test.
